# Going the Extra Mile: Effects of Discourse Context on Two Late Positivities During Language Comprehension

**DOI:** 10.1162/nol_a_00006

**Published:** 2020-03-01

**Authors:** Trevor Brothers, Eddie W. Wlotko, Lena Warnke, Gina R. Kuperberg

**Affiliations:** Tufts University, Medford, MA 02155; Moss Rehabilitation Research Institute, Elkins Park, Pennsylvania 19027; Tufts University, Medford, MA 02155; Tufts University, Medford, MA 02155

**Keywords:** prediction, event-related potential (ERP), P600, frontal positivity, discourse

## Abstract

During language comprehension, online neural processing is strongly influenced by the constraints of the prior context. Although the N400 event-related potential (ERP) response (300–500 ms) is known to be sensitive to a word’s semantic predictability, less is known about a set of late positive-going ERP responses (600–1,000 ms) that can be elicited when an incoming word violates strong predictions about upcoming content (late frontal positivity) or about what is possible given the prior context (late posterior positivity/P600). Across three experiments, we systematically manipulated the length of the prior context and the source of lexical constraint to determine their influence on comprehenders’ online neural responses to these two types of prediction violations. In Experiment 1, within minimal contexts, both lexical prediction violations and semantically anomalous words produced a larger N400 than expected continuations (*James unlocked the door/laptop/gardener*), but no late positive effects were observed. Critically, the late posterior positivity/P600 to semantic anomalies appeared when these same sentences were embedded within longer discourse contexts (Experiment 2a), and the late frontal positivity appeared to lexical prediction violations when the preceding context was rich and globally constraining (Experiment 2b). We interpret these findings within a hierarchical generative framework of language comprehension. This framework highlights the role of comprehension goals and broader linguistic context, and how these factors influence both top-down prediction and the decision to update or reanalyze the prior context when these predictions are violated.

## GENERAL INTRODUCTION

It is well established that during online language comprehension readers can extract relevant features of the prior context to facilitate the processing of new inputs. However, what constitutes “relevant context” can vary considerably—from single words in a semantic priming task to rich, multi-sentence discourse scenarios. In the present study, we investigated online neural responses when readers encountered predictable and unpredictable information, while systematically varying the length and semantic richness of the context that came before. By examining these responses in both early (300–500 ms) and late (600–1,000 ms) time windows, we hoped to provide a clearer picture of how and when these aspects of the broader linguistic context influence the neural mechanisms engaged in language comprehension.

One important tool for examining the influence of context on language comprehension is the event-related potential (ERP) technique. This method has revealed several distinct neural components that are differentially sensitive to manipulations of linguistic context. One of the most frequently investigated ERP components is the N400, which is a negative-going waveform observed between 300 and 500 ms after stimulus onset that is maximal over central-parietal electrode sites. During sentence comprehension, words that are semantically predictable in relation to their preceding context generate an N400 of smaller amplitude than words that are semantically unpredictable (Kutas & Hillyard, 1984) or anomalous (Kutas & Hillyard, 1980). The amplitude of the N400 evoked by an incoming word is thought to reflect the retrieval or access to its semantic features that have not already been predicted (Kuperberg, 2016; Kutas & Federmeier, 2011).

The N400 component has played an important role in understanding the relative influences of local and global contexts in facilitating online comprehension. Although some early, two-stage models of language comprehension argued for a delayed influence of global context on word processing (Rayner, Garrod, & Perfetti, 1992; Till, Mross, & Kintsch, 1988), a number of ERP studies have shown that discourse-based constraints can have an immediate influence on the amplitude of the N400 (Kuperberg, Paczynski, & Ditman, 2011; Van Berkum, Hagoort, & Brown, 1999; Van Berkum, Zwitserlood, Hagoort, & Brown, 2003). Moreover, these N400 effects are indistinguishable (in terms of latency and scalp topography) from N400 facilitation effects arising from local sentence contexts. These findings, in combination with similar results from eye-tracking while reading (Fitzsimmons & Drieghe, 2013), show that comprehenders can, in principle, rapidly combine information from both local and global contexts to help facilitate lexicosemantic processing (see Ledoux, Camblin, Swaab, & Gordon, 2006, for a review).

In contrast to the N400, much less is known about the context sensitivity of language-related ERP responses that peak after the N400 time window. Here we consider two such ERP components—the late frontal positivity and the late posterior positivity/P600—both positive-going waveforms that are detected on the scalp surface between 600 and 1,000 ms following word onset.

The late frontal positivity is maximal over frontal electrode sites and was first characterized as a response to plausible but unexpected words appearing in highly constraining sentence contexts (Federmeier, Wlotko, De Ochoa-Dewald, & Kutas, 2007; Kutas, 1993). For example, after a constraining context, *After proposing, he put the ring on her…*, an unexpected continuation (*dresser*) would not only elicit a larger N400 response, but also a larger late frontal positivity relative to the expected completion (*finger*). It has been variously proposed that this late frontal positivity response reflects the detection of a lexical prediction violation, the inhibition of an incorrectly predicted word (Federmeier et al., 2007; Kutas, 1993), and/or the incorporation of new unexpected information into a higher-level representation of meaning (Brothers, Swaab, & Traxler, 2015; DeLong, Quante, & Kutas, 2014; Federmeier, Kutas, & Schul, 2010; Kuperberg, Brothers, & Wlotko, 2020).

The late posterior positivity/P600, in contrast, is maximal over parietal and occipital sites, and was first characterized as an ERP response to words that are syntactically anomalous or structurally dispreferred (Hagoort, Brown, & Groothusen, 1993; Osterhout & Holcomb, 1992). This initial association between the P600 and syntactic processing was later revised when it became clear that syntactically correct sentences with anomalous semantic interpretations can also elicit the effect. For example, Kuperberg, Sitnikova, Caplan, and Holcomb (2003) showed that semantically anomalous sentences (e.g., *Every morning for breakfast the eggs would eat…*) produced a robust late posterior positivity/P600 effect relative to plausible control sentences (*Every morning for breakfast the boys would eat…*). It is important to note that unlike the late frontal positivity effect, the late posterior positivity/P600 effect is typically produced by continuations that are perceived as semantically impossible given the constraints of the prior context (Kuperberg, 2007; van de Meerendonk, Kolk, Vissers, & Chwilla, 2010; see Van Petten & Luka, 2012 for a review of the early literature). This component has been linked to the detection of conflict between competing interpretations during language comprehension (Kim & Osterhout, 2005; Kuperberg, 2007; Paczynski & Kuperberg, 2012; van de Meerendonk, Kolk, Chwilla, & Vissers, 2009), second-pass reanalysis (van de Meerendonk et al., 2010), and/or prolonged attempts to make sense of the input (Kuperberg, 2007).

We have recently proposed that these two late positivities and the N400 can be understood within a single theoretical framework, by appealing to a hierarchical generative model of language comprehension (Kuperberg, 2016; Kuperberg et al., 2020; Kuperberg & Jaeger, 2016). Within this framework, the comprehender draws upon a *generative model—*a network of stored linguistic and nonlinguistic representations that they believe are relevant to achieve their current goal. Information within this network is organized across multiple representational levels, which include semantic and syntactic features, as well as *event structures* that describe the semantic-thematic roles of individual propositions (see Dowty, 1989; Jackendoff, 1987). If the comprehender’s overarching goal is deep comprehension, then, at the highest level of the network, they will establish a *situation model*. Rather than simply encoding a surface-level representation of the text, this situation model constitutes a high-level representation of the full set of events being conveyed within the prior discourse, including the referential, spatial, temporal, motivational, and causal coherence relationships that link them together (Van Dijk & Kintsch, 1983; Zwaan & Radvansky, 1998).

Unlike earlier models of text/discourse comprehension (e.g., Kintsch, 1988), this hierarchical generative framework is both generative and anticipatory. At any particular time, the comprehender may have high-level “hypotheses” at the level of the situation model, and, to test these hypotheses, propagate probabilistic predictions down to lower levels of the hierarchy (“top-down prediction”). Because new bottom-up information flows up the network, any information that has already been predicted is “explained away,” and any unpredicted information (prediction error) is passed up the hierarchy to update hypotheses at higher levels. In the brain, this type of message-passing scheme is known as *predictive coding* (Friston, 2005; Mumford, 1992; Rao & Ballard, 1999).

We refer to the full generative model that is deployed during deep comprehension (the situation model and levels of the network below it) as the *communication model*. Because this communication model specifies the comprehender’s assumptions about the current speaker and communicative environment (cf. Frank & Goodman, 2012), it places constraints over what sorts of inputs can (or cannot) be successfully incorporated at different levels of the hierarchy. For example, a comprehender’s communication model may incorporate the assumption that the communicator will describe events that are possible in the real world, placing constraints over the inputs that can be incorporated into the situation model.

As discussed by Kuperberg et al. (2020), within this hierarchical generative framework, the N400 reflects the process of accessing semantic features associated with the incoming word that have not already been predicted from the prior context (semantic prediction error). The late frontal positivity is hypothesized to reflect shifts in belief at the level of the situation model in order to successfully incorporate new unanticipated information. Finally, the late posterior positivity/P600 is triggered when the bottom-up input is incompatible with the communication model, and therefore cannot be successfully incorporated at a particular level of the hierarchy. In the case of the P600 evoked by semantic anomalies, this component is triggered by a failure to incorporate the new input at the level of the situation model. This results in prolonged attempts to make sense of the input through second-pass reanalysis or repair, and possibly through modifying the constraints of the communication model itself (adaptation).

### The Role of Context and the Present Study

Within the hierarchical generative framework described above, both the late frontal positivity and the “semantic” late posterior positivity/P600 are thought to be generated by mismatches between the bottom-up input and the content/constraints present at the level of the situation model. Thus, according to this account, if no situation model has been established—that is, if the goal is not deep comprehension—then these two ERP responses should not be elicited, regardless of the predictability or plausibility of the bottom-up input. Clearly, many factors can determine whether we engage in deep comprehension and establish a situation model. These include the explicit task given to the reader, their internal motivation, and, of most relevance to the present study, the nature of the preceding context (see Zwaan & Radvansky, 1998, for discussion). For example, contexts that introduce characters, goals, and coherence relationships between events will encourage comprehenders to establish a situation model—that is, to engage in deep comprehension. In contrast, following a short, semantically impoverished context, comprehenders may fail to establish a situation model, with potential consequences for the elicitation of these late positive components.

Although this issue has not been investigated in detail, there is some preliminary evidence supporting a link between the two late ERP responses and the nature of the prior context. For example, although the late frontal positivity is typically observed in response to unexpected words within extended single-sentence (DeLong, Urbach, Groppe, & Kutas, 2011) and multi-sentence discourse scenarios (Kuperberg et al., 2020), this effect has not been observed in simple semantic priming paradigms. Although semantically associated primes produce clear N400 reductions on expected target words (*hot* − *cold*) (Bentin, McCarthy, & Wood, 1985; Rugg, 1985), there is no enhancement of the late frontal positivity to unexpected targets (e.g., *hot* − *brown*), even when the broader environment encourages participants to predict (Holcomb, 1988; Lau, Holcomb, & Kuperberg, 2013).

The nature of the prior context has also been linked to the presence of the late posterior positivity/P600 following semantic anomalies (see Kuperberg, 2007, for discussion). In a review of the early literature, Szewczyk and Schriefers (2011) noted that semantic anomalies presented in impoverished sentence contexts often fail to show a late posterior positivity/P600 (e.g., Ainsworth-Darnell, Shulman, & Boland, 1998; Friederici, Pfeifer, & Hahne, 1993; Friederici, Steinhauer, & Frisch, 1999; Gunter, Friederici, & Hahne, 1999; Osterhout & Nicol, 1999; see also Kos, Vosse, van den Brink, & Hagoort, 2010; Lau, Namyst, Fogel & Delgado, 2016; Li, Shu, Liu, & Li, 2006), while studies with longer introductory contexts (three or more content words preceding a violation) often do show a posterior late positivity/P600, at least based on an informal inspection of the ERP waveforms (for early examples, see Ganis, Kutas, & Sereno, 1996; Kutas & Hillyard, 1980, 1983; Münte, Heinze, Matzke, Wieringa, & Johannes, 1998). Perhaps the most direct illustration of this relationship between context and the late posterior positivity/P600 comes from an auditory comprehension study by Nieuwland and Van Berkum (2005). In this study, when participants encountered a semantic anomaly at the beginning of a discourse (*Next, the woman told the suitcase…*), this anomaly produced a robust N400 effect. However, when the same anomaly was placed at the end of an otherwise coherent narrative, it produced no N400 and a greatly enhanced late posterior positivity/P600 effect instead.

Although these previous studies provide some evidence that the length and/or constraint of a prior context can play a role in modulating both the late frontal positivity and the late posterior positivity/P600, the hypothesized link between context and these two late ERP responses has not been systematically investigated. Specifically, it remains unclear what contextual factors may play a role in modulating these effects, including: (a) the amount of prior context, (b) its degree of constraint for an upcoming word, and/or (c) the source of lexical constraint (originating from a local context vs. a rich global context). By examining these questions, we hope to shed light on the functional significance of these two late positive effects, as well as the neurocognitive processes that are engaged when readers encounter unpredicted linguistic inputs.

Our starting point was a recent study by Kuperberg et al. (2020), who dissociated the N400, the late frontal positivity, and the late posterior positivity/P600 effects in a single study during multi-sentence discourse comprehension. In that study, participants read and monitored the coherence of three-sentence scenarios, some of which were highly predictive of a particular direct object noun.

*The lifeguards received a report of sharks right near the beach. Their immediate concern was to prevent any incidents in the sea. Hence, they cautioned the…*

1a) …*swimmers* (Expected)

1b) …*trainees* (Unexpected)

1c) …*drawer* (Anomalous)

This study showed a clear dissociation between the amplitude of the N400, which was primarily sensitive to lexical probability, and the two late positivities that were produced by words that violated prior predictions. Critically, the late frontal positivity was observed primarily to plausible but unexpected words (*trainees*), whereas semantic anomalies (*drawer)* that yielded an impossible interpretation, instead evoked the late posterior positivity/P600.

In the present set of experiments, we used a parallel experimental design that systematically manipulated the length of the prior context and the source of lexical constraint to investigate their role in eliciting these three ERP components. In Experiment 1, participants read minimal sentence contexts in which the constraint of the context was provided by a single verb in the local context (*Susan swept the…*), and ERPs were recorded to expected, unexpected, and anomalous continuations (Experiment 1, single sentence). In Experiment 2a, ERP responses were recorded to these same *locally constraining* sentences when presented as part of a longer three-sentence scenario. In Experiment 2b, ERP responses were recorded to a separate set of *globally constraining* three-sentence scenarios (a subset of those used in Kuperberg et al., 2020), in which lexical constraint at the critical word depended on the entire preceding discourse. In this way, we separately examined how the length of the preceding context (Experiment 1 vs. 2a) and its source of lexical constraint (Experiment 2a vs. 2b) might influence how readers process contextually unexpected information during online comprehension.

## EXPERIMENT 1

### Introduction

In Experiment 1, we contrasted ERP responses to expected, unexpected, and anomalous critical words, following prior studies that have dissociated the late frontal and posterior positivities. However, rather than using multi-sentence discourse contexts (DeLong et al., 2014; Kuperberg et al., 2020), we used very simple three-word contexts in which constraint was determined purely by the lexical properties of a single verb (“unlocked” in the examples below):

*James unlocked the…*

2a) …*door* (Expected)

2b) …*laptop* (Unexpected)

2c) *…gardener* (Anomalous)

A large body of previous work in sentence comprehension suggests that words are recognized more rapidly and produce smaller N400 amplitudes when they are predictable given the preceding context (Kutas & Hillyard, 1984; Rayner & Well, 1996). We therefore predicted that, relative to the two other conditions, the amplitude of the N400 would be reduced on critical words that were highly expected given the constraints of the prior verb.

Our main question was whether the late frontal and posterior positivities would be produced by words that violated these short, lexically constraining sentence contexts. As noted in the General Introduction, although the late frontal positivity is reliably observed to unexpected words in longer sentence and discourse contexts, this effect is not typically observed in single word-priming paradigms. In addition, there is evidence that the late posterior positivity/P600 evoked by semantic anomalies is attenuated or even absent when the preceding context is short (Kuperberg, 2007; Lau et al., 2016; Nieuwland & Van Berkum, 2005; Szewczyk & Schriefers, 2011). In the current experiment, we asked whether these two late positivities would be observed in short, relatively impoverished sentence contexts, which, nonetheless, still constrained for a particular upcoming word.

As discussed in the General Introduction, there has been some debate about the neurocognitive mechanisms underlying these two positivities, with some accounts emphasizing their role in detecting violations at lower levels of representations, and others, including the generative framework outlined above, emphasizing their role in higher-level comprehension. The results of this experiment can help arbitrate between these broad accounts. Specifically, if a robust late frontal positivity is elicited by unexpected continuations following short but lexically constraining contexts (*James unlocked the laptop*), this would suggest that violations of lexical constraint are sufficient for triggering this ERP response. Similarly, if a robust late posterior positivity is observed to anomalous continuations (*James unlocked the gardener*), this would suggest that outright violations of a verb’s selectional restrictions are sufficient for triggering this response. If, however, no late positivities are observed in Experiment 1, this would suggest that, rather than simply reflecting the violation of lexical predictions, these ERP effects may, at least in part, reflect activity at a higher levels of representation.

#### Materials

To generate sentence materials for this ERP study, we first needed to identify a set of verbs that were predictive of a particular upcoming noun (e.g., *unlocked the door, dimmed the lights, raked the leaves)*. To accomplish this, we first collected a set of 617 transitively biased verbs from a variety of sources (Levin, 1993; Paczynski & Kuperberg, 2011, 2012). We then carried out a behavioral cloze norming task to determine which of these verbs were the most lexically constraining. Participants, recruited from Amazon’s Mechanical Turk, were asked to read a set of short active sentences containing a proper name, the verb, and a determiner (e.g., *James unlocked the…*). For each sentence, participants filled in the first continuation that came to mind. After excluding participants who failed to follow instructions, a minimum of 90 cloze responses were obtained for all verbs. The lexical constraint of each verb was determined by calculating the proportion of participants providing the most common completion for each context.

Based on these cloze norms, we selected 87 verbs that constrained for a particular noun. The average constraint of these verbs was 63% (standard deviation [SD]: 15%, range: 99%–36%). For each verb, we selected three nouns: an expected noun that was always the most common completion provided in the cloze test, an unexpected noun that was still plausible, and a semantically anomalous noun that violated the selection constraints of the preceding verb (*James unlocked the door/laptop/gardener…*). The unexpected and anomalous nouns were closely matched in cloze probability (both less than 1%). Moreover, unexpected and anomalous nouns were lexically identical, with the same words being counterbalanced across sentence contexts in a Latin square design (*Judy alerted the police/gardener/laptop…*; see supplementary appendix [https://www.mitpressjournals.org/doi/suppl/10.1162/nol_a_00006] for additional examples). On average, the expected nouns were slightly shorter and more frequent than the unexpected/anomalous nouns (*expected*: 4.9 characters, *unexpected*: 6.6, *anomalous*: 6.6; *expected*: 3.7 log per million, *unexpected*: 2.6, *anomalous*: 2.6, SUBTLEX-US frequency; Brysbaert & New, 2009).

Using these noun-verb pairings, we generated a set of short, simple active sentences. Each sentence included a proper name, a verb, and a determiner, with the critical noun always appearing as the fourth word of the sentence (*Beth raked the leaves*). We then asked a new group of 36 participants (12 per list) to rate the plausibility of each item on a seven-point scale (ranging from “makes perfect sense” to “makes no sense at all”). As expected, there were clear differences in plausibility between the three conditions (*expected*: mean = 6.8, SD = 0.3, *unexpected*: mean = 5.8, SD = 1.1, *anomalous*: mean = 1.8, SD = 0.6; all pairwise *p* < .001).

For the main ERP experiment, we added an additional three to seven words to each sentence, which were held constant across conditions, ensuring that the critical noun was never sentence-final (e.g., *Beth raked the leaves out back in the yard*). The three high constraint conditions described above (*James unlocked the door/laptop/gardener…*) and an additional low constraint filler condition (*Emma confiscated the laptop…*) were counterbalanced across four lists. Items in this fourth filler condition contained verbs that were not highly predictive of an upcoming noun (constraint = 21%, SD = 9%) and which could also continue with a plausible or anomalous continuation. These fillers ensured that not all of the sentences were highly constraining, as in Kuperberg et al. (2020). Because of this counterbalancing scheme, participants saw 21 to 22 items in each of our three conditions of interest, which were randomly interspersed with 154 filler sentences with a similar structure. Thus, participants saw 220 trials in total, with an equal number of sentences with high- and low-constraint verbs and an equal number of sentences with plausible and anomalous object nouns.

#### Participants

We report data from 30 participants (16 female, mean age = 22 years). Data from four additional participants were excluded—one due to excessive EEG artifact and three due to accuracies below 75% in the acceptability judgment task (see subsequent text). All participants were recruited from Tufts University and the surrounding community. They were screened on the basis of the following exclusion criteria: significant exposure to any language other than English before the age of 5, history of psychiatric or neurological diagnoses or injury, and the use of psychoactive medication within the preceding 6 months. Participants were right-handed as assessed by the Edinburgh Handedness Inventory. They provided written informed consent, were paid for their time, and all protocols were approved by Tufts University Social, Behavioral, and Educational Research Institutional Review Board.

#### Procedure and preprocessing

Participants sat comfortably in a dimly lit room, approximately 100 cm from the computer screen. After being prepared for EEG, they performed a short practice session, followed by four experimental blocks. On each trial, participants first saw a central fixation cross, and a sentence was then presented, one word at a time in the center of the screen. Each word appeared for 450 ms with a blank 100-ms interstimulus interval between words. Participants were asked to read each sentence silently for comprehension, and, at the end of each trial, to judge whether or not the preceding sentence “made sense,” indicating their decision by pressing one of two keys. The entire experiment lasted approximately 1 hour.

Throughout the experiment, EEG was recorded from the scalp using 32 active electrodes (with a modified 10/20 system montage), using a Biosemi Active-Two acquisition system. Signals were digitized at 512 Hz and a passband of DC - 104 Hz. The EEG was referenced offline to the average of the right and left mastoids, and a 0.1 to 30 Hz bandpass filter was applied. The EEG was then segmented into epochs (−100 ms to 1,000 ms), time-locked to the onset of the critical noun. Epochs containing artifacts such as blinks, eye movements, or muscle artifacts were rejected prior to analysis. On average, there were 18 trials per condition following artifact rejection (range: 10–22), with no significant differences in artifact rejection rates across conditions, *F*(2,58) = 0.8, *p* = .48.

#### Statistical analysis strategy

EEG data were averaged for each subject and condition using a −100-ms prestimulus baseline. EEG amplitudes were then averaged across electrode sites within specific spatiotemporal regions of interest that were selected a priori based on a previous study using a similar design (Kuperberg et al., 2020). The N400 was operationalized as the average voltage across electrode sites within a central region (Cz, CPz, C3/4, CP1/2) between 300 and 500 ms. The late frontal positivity was operationalized as the average voltage across electrode sites within a prefrontal region (FPz, AFz, FP1/2, AF3/4) between 600 and 1,000 ms, and the late posterior positivity/P600 as the average voltage within a posterior region (Pz, Oz, P3/4, O1/2), also between 600 and 1,000 ms. Within each spatiotemporal region, we first conducted an omnibus analysis of variance (ANOVA; three levels of Condition), which was then followed up by pairwise comparisons of interest.

### Results

#### Behavioral results

In the final sample of participants, behavioral accuracy was generally high (89%). Participants were more accurate in judging the plausibility of sentences with expected critical words (95%), than those with unexpected (85%) or anomalous (86%) critical words.

#### ERP results

##### N400 (300–500 ms, Central region).

In the N400 spatiotemporal region of interest, we observed a main effect of Condition, *F*(1, 29) = 17.3, *p* < .001, which was driven by a reduction in N400 amplitudes for expected versus unexpected critical words, *t*(29) = −4.84, *p* < .001. N400 amplitudes to unexpected and anomalous critical words did not differ significantly from one another, *t*(29) = 0.90, *p* = .35.

##### Late frontal positivity (600–1,000 ms, Prefrontal region).

Unlike for the N400, there was no main effect of Condition on the late frontal positivity, *F*(1, 29) = 0.64, *p* = .53. Consistent with this null effect, a planned pair-wise comparison revealed no significant differences between expected and unexpected critical words, *t*(29) = 0.97, *p* = .34.

##### Late posterior positivity/P600 (600–1,000 ms, Posterior region).

Similar null effects were observed on the late posterior positivity/P600. Again, we observed no significant main effect of Condition, *F*(1, 29) = 0.2, *p* = .81, and a planned pair-wise comparison confirmed that there was no significant difference between expected and anomalous critical words, *t*(29) = 0.22, *p* = .83.

### Discussion

In Experiment 1, participants read short active sentences and judged whether they made sense while ERPs were recorded from the scalp. Our goal was to assess the effects of minimal sentential contexts on neural responses to expected, unexpected, and semantically anomalous words. As expected, the amplitude of the N400 evoked by predictable nouns (*James unlocked the door*) was smaller than that evoked by unpredictable but plausible continuations (*laptop*) or anomalous continuations (*gardener*). This finding is consistent with many previous findings relating cloze probability and the N400 (Kutas & Hillyard, 1984), as well as with studies demonstrating facilitated semantic access to words preceded by semantically associated contexts (Camblin, Gordon, & Swaab, 2007; Li et al., 2006).

**Figure 1.**
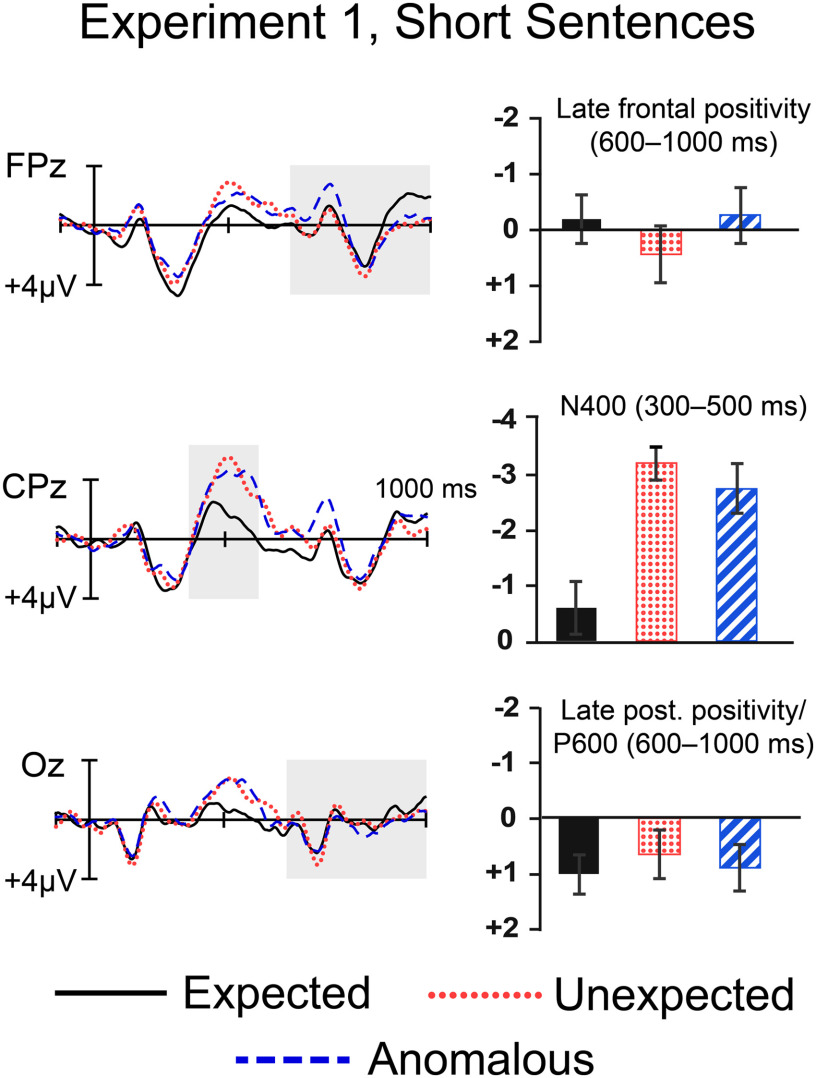
Grand-average event-related potentials (ERPs) for the three conditions, plotted at three midline electrode sites. In this and subsequent figures, negative is plotted up, and all waveforms were low-pass filtered at 15 Hz for presentation purposes. Bar graphs to the right show average voltages within each spatiotemporal region of interest (see text) with ±1 standard error of the mean (SEM) error bars, calculated within-subject (Morey, 2008).

Despite these clear differences on the N400, we saw no significant effects following the N400 time window (600–1,000 ms), either at frontal or posterior electrode sites (Figure 1). This finding differs from the results of a recent ERP study using rich discourse contexts (Kuperberg et al., 2020), which demonstrated both late positivities in sentences with a similar syntactic structure and similar levels of lexical constraint. In terms of the late frontal positivity, this result suggests that the presence of a lexical prediction violation alone is not sufficient to elicit the effect. In terms of the late posterior positivity/P600, it suggests that the presence and successful detection of a semantic anomaly (a selectional restriction violation) is not sufficient to elicit this effect. In the absence of a prior context, participants in this experiment may have failed to establish a situation model. Instead of engaging in deep comprehension, they may have achieved high behavioral accuracy in this task by attending primarily to the surface level of the text, monitoring for a match or mismatch between the selectional constraints of the verb and the semantic features of the critical noun.

Although the results of Experiment 1 are suggestive, they leave open the question of exactly *what* features of the prior context are necessary for eliciting these two late ERP responses. One possibility is that the presence of *any* discourse context—even one that is nonconstraining—is sufficient to encourage participants to establish a situation model and engage in deep comprehension (Van Dijk & Kintsch, 1983; Zwaan & Radvansky, 1998). Another possibility is that comprehenders require a rich and globally constraining linguistic context in order to elicit these two ERP effects. For example, in the high constraint contexts used by Kuperberg et al. (2020), the lexical constraint at the point of the critical noun was derived from the entire preceding context, rather than just the immediately preceding verb. This type of rich, globally constraining context would lead comprehenders to build a *rich* situation model that constraints for an upcoming event, and it is possible that the late frontal and posterior positivities are elicited only within these types of rich, globally constraining linguistic contexts.

Our goal in Experiment 2 was to help adjudicate between these two possibilities.

## EXPERIMENT 2

### Introduction

In Experiments 2a and 2b, we again measured ERPs to expected, unexpected, and anomalous nouns. This time, however, they appeared in two-sentence discourse contexts. We varied these contexts such that the source of lexical constraint came either from only the preceding verb (*locally constraining*: Experiment 2a) or from the entire discourse context (*globally constraining*: Experiment 2b).

In Experiment 2a, we presented the same locally constraining sentences used in Experiment 1. However, these sentences were now presented at the end of two-sentence discourse contexts. The events described in these two introductory sentences were generally vague, and they were not strongly associated with the event described in sentence three. Thus, just as in Experiment 1, any lexical constraints at the point of the critical noun were determined primarily by the lexical properties of the preceding verb. We refer to these items as *locally constraining* discourse scenarios.

Locally constraining: *He was thinking about what needed to be done on his way home. He finally arrived. James unlocked the (door/laptop/gardener)….*

In Experiment 2b, we used rich, globally constraining discourse scenarios, which were a subset of the high constraint items used by Kuperberg et al. (2020). Unlike the items in Experiment 2a, these discourse scenarios were semantically rich and interconnected, and the expected event in the final sentence followed naturally from the set of events described in sentences one and two. Notably, the verb in the final sentence was always nonconstraining in isolation (e.g., *cautioned*). Thus, these scenarios were also lexically constraining at the point of the critical noun, but this constraint stemmed from the entirety of the preceding discourse context rather than the preceding verb. We refer to these items as *globally constraining* discourse scenarios.

Globally constraining: *Tim really enjoyed baking apple pie for his family. He had just finished mixing the ingredients for the crust. To proceed, he flattened the (dough/foil/onlookers)….*

We were careful to match the strength of lexical constraint between the locally constraining contexts (used in Experiment 2a) and the globally constraining contexts (used in Experiment 2b), as quantified by our cloze norms. This enabled us to examine how the nature of the prior context (locally vs. globally constraining) influenced the processing of expected, unexpected, and anomalous sentence continuations. Specifically, by contrasting Experiment 1 with Experiment 2a, we were able to isolate the influence of the presence of prior context, and by comparing the results of Experiments 2a and 2b, we were able to isolate the effects of global versus local constraint on the N400, late frontal positivity, and late posterior positivity/P600.

As noted in the General Introduction, there is a consensus that extrasentential context can immediately influence the difficulty of accessing the semantic features of incoming words, as indexed by the N400 (Kuperberg et al., 2011; Van Berkum et al., 1999, 2003). However, there have been no ERP studies that have directly compared the effects of local and global context when the probability of the critical word is matched across conditions. In one behavioral study addressing this question (Fitzsimmons & Drieghe, 2013), readers showed similar effects of local and global contextual constraints on eye-movement behavior. Specifically, reading times and word skipping rates were equivalent on words that were globally predictable (*I looked up after hearing a chirping noise. I saw a bird…*) and locally predictable (*I looked up to the sky. I saw a feathered bird…*), with evidence of facilitation in both conditions relative to an unpredictable baseline. Therefore, assuming that early eye-movement measures and the N400 both reflect the difficulty of lexicosemantic retrieval, we predicted similar N400 facilitation effects on expected critical words in both locally constraining discourse contexts (Experiment 2a) and globally constraining discourse contexts (Experiment 2b).

Of primary interest was how the prior discourse context would influence modulation of the two late post-N400 positivities. As discussed earlier, one possibility was that the short, impoverished contexts presented in Experiment 1 encouraged participants to engage in a shallow processing strategy. On this account, participants relied purely on matching the lexical-thematic properties of the verb with the semantic and syntactic features of the noun in order to provide acceptability judgments. If this is the case, then the addition of two-sentence contexts in Experiment 2a should encourage readers to establish a basic situation model and re-engage in deep comprehension, which in turn could enhance the amplitude of these two late positive components.

Alternatively, it may be that the critical factor for eliciting these late positivities is the establishment of a rich and interconnected situation model that globally constrains for a specific upcoming event. On this account, only a rich and globally constraining context would afford participants the opportunity to build this type of situation model, and these two late positivities should only emerge in Experiment 2b.

Finally, within the hierarchical generative framework outlined in the General Introduction, these two manipulations may have dissociable effects on the two late positivities. It may be the case that even an impoverished context is sufficient to establish a full communication model, with constraints over what sorts of events can be successfully incorporated. If this is the case, then any input that is incompatible/conflicts with these constraints would lead to a failure to incorporate the input into the situation model, evoking a late posterior positivity/P600. In contrast, if the late frontal positivity indexes a large update or reinterpretation of the prior situation model, then this ERP response should be observed only in Experiment 2b in which the lexical constraint of the critical word stems from a rich, globally constraining discourse.

### Methods

#### Materials

For this experiment we used two different sets of materials. The first was a set of locally constraining three-sentence discourse scenarios that we developed based on the single sentences used in Experiment 1, which we refer to as Experiment 2a. The second was a set of globally constraining materials, which we refer to as Experiment 2b.

To develop our locally constraining discourse scenarios (Experiment 2a), a two-sentence introductory context was added to each sentence used in Experiment 1. These introductions were written to be plausible but relatively unassociated with the expected event occurring in the final critical sentence. Thus, just as in Experiment 1, the predictability of the expected critical noun was driven purely by the lexical properties of the preceding verb (*The group always split up their responsibilities evenly. Everyone did their share. Charlotte swept the floor…*). To confirm that these discourse contexts did not alter the lexical constraint of the critical noun, we conducted a second cloze norming study on these discourse materials using a new pool of participants, using the same procedures as those described under Experiment 1. After exclusions, a minimum of 50 cloze responses were collected for each discourse context. This confirmed that the cloze probability of the expected critical noun did not differ between Experiment 1 and Experiment 2a (Experiment 1, single sentences: 63%, SD = 16%; Experiment 2a, locally constraining discourse contexts: 63%, SD = 17%, *t* < 1).

For the globally constraining discourse materials (Experiment 2b), we used a different set of discourse scenarios—a subset of the “high constraint contexts” used by Kuperberg et al. (2020). In these scenarios, the final sentence was similar in structure to those used in the locally constraining materials described earlier, but they included verbs that, when presented in isolation, did not constrain strongly for a particular noun (average constraint = 16%, e.g., *worried*, *designed*, *greeted*). Unlike the locally constraining contexts used in Experiment 2a, these sentences were preceded by a rich two-sentence discourse context. These contexts often contained links establishing coherence across sentences, including co-reference, temporal relationships, intentionality, and causation (Zwaan & Radvansky, 1998). Thus, in combination with the first few words of the final sentence, these rich discourse contexts made a particular upcoming critical noun highly predictable (e.g., *The aircraft was behaving strangely. Something on the control panel seemed off. Certainly, this worried the pilot…*).

For both Experiments 2a and 2b, each constraining context was again paired with three possible continuations: an expected noun, an unexpected but plausible noun, or a semantically anomalous noun. As before, the unexpected continuations were always lexically unpredictable (<1%) but plausible, and both the unexpected and anomalous nouns were counterbalanced across items. As in Experiment 1, the critical nouns were never sentence-final and the words immediately following the critical word were always held constant across conditions (see supplementary appendix [https://www.mitpressjournals.org/doi/suppl/10.1162/nol_a_00006] and Kuperberg et al., 2020, for additional information).

Offline cloze ratings confirmed that the lexical constraint of the locally constraining discourse contexts in Experiment 2a and the globally constraining contexts used in Experiment 2b were matched (Experiment 2a, locally constraining: 63%, SD = 16%; Experiment 2b, globally constraining: 66%, SD = 10%, *t*(163) = 1.44, *p* = .15). Thus, these two stimulus sets differed in the *source* of lexical constraint (Experiment 2a: preceding verb; Experiment 2b: global context), but not in the degree of lexical constraint just prior to the critical noun.

In addition, we also wished to verify our intuition that the globally constraining contexts were more semantically rich and interconnected than the locally constraining contexts in Experiment 2a. Therefore, we conducted an additional rating study with a new group of 15 participants recruited from Amazon’s Mechanical Turk. Participants read the expected version of each three-sentence scenario (truncated after the critical word), and they were asked to rate (1–5) how “associated or connected the final sentence is to the previous context” (5 = *very connected*; 1 = *very disconnected*). As expected, there were large differences in “connectedness” ratings across the two stimulus sets (Experiment 2a: mean = 2.7, SD = 0.8; Experiment 2b: mean = 4.7, SD = 0.2). In addition, although each scenario always contained two introductory sentences, these two sentences had more words on average in the globally constraining scenarios (Experiment 2a: mean = 14.5, SD = 3; Experiment 2b: mean = 21.7, SD = 4).

In the ERP experiment, these two sets of experimental stimuli were presented in a randomized order in the same experimental session. In addition to these 174 experimental trials (29 per condition), participants also saw 116 filler scenarios, which balanced the proportion of plausible and implausible trials across the experiment.

#### Participants

In Experiment 2, a new group of 30 participants was recruited from Tufts University and the surrounding community using the same screening criteria used in Experiment 1 (21 female, mean age = 23 years). None of these participants were excluded due to excessive artifact or low comprehension accuracy. All participants provided written informed consent and were paid for their time.

#### Procedure

Participants read each three-sentence discourse scenario, while EEG was recorded from the scalp. The first two sentences of each scenario were presented in full, and participants pressed a button when they were ready to continue to the next sentence. The final sentence was then presented one word at a time, with the same timing as in Experiment 1. At the end of each trial, participants indicated via button-press whether the preceding scenario made sense or not. In addition, on 10% of trials, participants were given a True/False comprehension question to ensure that they were attending to all three sentences of the preceding discourse. The experimental stimuli were split into 10 blocks, and the entire recording session lasted approximately 1.5 hours. EEG recording, filtering, and artifact rejection parameters were identical to those for Experiment 1. On average, following artifact rejection, 23 trials remained in each condition (range: 15–26), and there were no significant differences in artifact rejection rates across conditions, *F*(5,145) = 1.5, *p* = .21.

#### Analysis strategy

**Figure 2.**
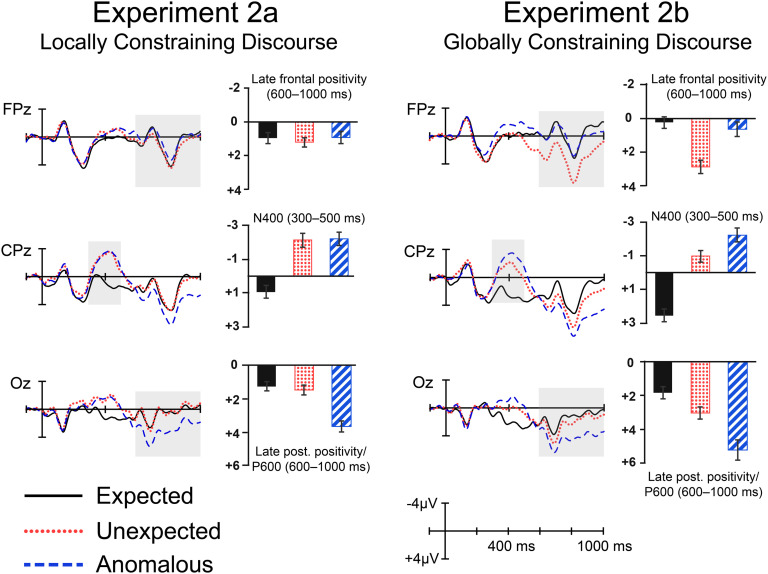
Grand-average event-related potentials (ERPs) in Experiments 2a and 2b, plotted at three midline electrode sites. The bar graphs show average voltages within each spatiotemporal region of interest (see text) with ±1 standard error of the mean (SEM) error bars, calculated within-subject.

We analyzed Experiments 2a and 2b using the same strategy as that described for Experiment 1 (three spatiotemporal regions corresponding to the N400, the late frontal positivity, and the late posterior positivity/P600; see Figure 2). Because the critical nouns in Experiment 2a (locally constraining contexts) and Experiment 2b (globally constraining contexts) differed in sentence position, the main effect of “Experiment” was not particularly informative. Therefore, our analyses focused first on the main effect of Condition within each experiment, followed by cross-experiment comparisons to determine whether these main effects of Condition differed across experiments (Experiment 1 vs. Experiment 2a, and Experiment 2a vs. Experiment 2b).

### Results

#### Behavioral results

As in Experiment 1, accuracy in the acceptability judgment task was high, both in the locally constraining (91%) and the globally constraining discourse contexts (94%). Comprehension question accuracy was also high (93%), suggesting that participants were carefully attending to all three sentences of the discourse scenarios.

#### ERP results: Experiment 2a (locally constraining discourse contexts)

##### N400 (300–500 ms, Central region).

On the N400, we saw a main effect of Condition, *F*(2, 58) = 21.1, *p* < .001, reflecting a smaller N400 to expected critical words than to both unexpected, *t*(29) = −5.58, *p* < .001, and anomalous, *t*(29) = −5.91, *p* < .001, critical words. The amplitude of the N400 did not differ between the unexpected and anomalous critical words, *t*(29) = −0.15, *p* = .88.

##### Late frontal positivity (600–1,000 ms, Prefrontal region).

Just as in Experiment 1, there was no main effect of Condition on the late frontal positivity, *F*(2, 58) = 0.22, *p* = .79, with planned comparisons confirming that there was no difference between unexpected and anomalous critical words, *t*(29) = 0.58, *p* = .57.

##### Late posterior positivity/P600 (600–1,000 ms, Posterior region).

In contrast, we observed clear differences across conditions in the amplitude of the late posterior positivity/P600, *F*(1,29) = 19.7, *p* < .001. Unlike in Experiment 1, the late posterior positivity/P600 was larger to semantically anomalous than expected critical words, *t*(29) = 4.75, *p* < .001. The amplitude of the late posterior positivity/P600 did not differ between the unexpected and expected critical words, *t*(29) = 0.56, *p* = .85.

#### ERP results: Experiment 2b (rich, globally constraining contexts)

##### N400 (300–500 ms, Central region).

Just as in Experiments 1 and 2a, there was a main effect of Condition, *F*(2, 58) = 44.4, *p* < .001, reflecting a smaller N400 to expected than to unexpected, *t*(29) = −7.21, *p* < .001, and anomalous, *t*(29) = −8.26, *p* < .001, critical words. Although smaller in magnitude, there were also significant differences in the N400 between the unexpected and anomalous critical words, *t*(29) = −2.50, *p* = .02, which is generally consistent with prior findings (see Kuperberg et al., 2020, for further discussion).

##### Late frontal positivity (600–1,000 ms, Prefrontal region).

Unlike in Experiment 2a, in rich, globally constraining contexts, the late frontal positivity did show significant differences across the three conditions, *F*(2, 58) = 13.4, *p* < .001, due to a larger late frontal positivity on unexpected than expected critical words, *t*(29) = 5.33, *p* < .001. There was no difference in this component between the semantically anomalous and expected words, *t*(29) = 0.72, *p* = .48.

##### Late posterior positivity/P600 (600–1,000 ms, Posterior region).

**Figure 3.**
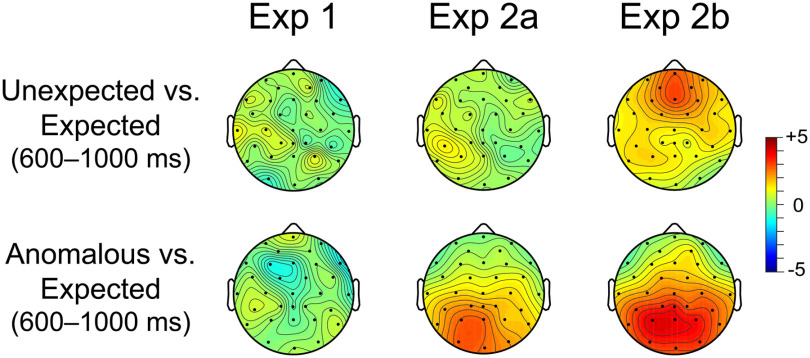
Topographic difference maps demonstrating the magnitude (μV) and scalp distribution of late event-related potential (ERP) effects (600–100 ms) across the three experiments.

Finally, in these rich globally constraining contexts, just as in Experiment 2a, we observed clear differences across the three conditions on the late posterior positivity/P600, *F*(1, 29) = 14.5, *p* < .001, again due to a significantly larger late posterior positivity/P600 on anomalous than expected critical words, *t*(29) = 4.55, *p* < .001. There was also a difference in this spatiotemporal region between unexpected nouns and expected critical word, *t*(29) = 3.52, *p* = .001, although this likely reflects the widespread late frontal positivity evoked by unexpected words, which was also visible over some posterior electrodes (Figure 3).

#### Comparisons across Experiments

##### Comparison between Experiment 1 and Experiment 2a.

Although the pattern of facilitation on the N400 and the null effects on the late frontal positivity were similar across Experiment 1 and Experiment 2a, the effect of semantic anomaly on the late posterior positivity/P600 differed across these two experiments. Specifically, although semantically anomalous words did not produce a late posterior positivity/P600 effect in Experiment 1 (short sentence contexts), this effect was present in Experiment 2a, when these same anomalies appeared in discourse contexts (see Figure 3).

To determine whether these between-experiment differences were statistically reliable, we directly compared all three effects—the N400 effect (unexpected minus expected), the late frontal positivity effect (unexpected minus expected), and the late posterior positivity/P600 effects (anomalous minus expected) between the two experiments. This between-subject analysis confirmed a significant difference between Experiment 1 and Experiment 2a in the magnitude of the anomaly effect on the late posterior positivity/P600 (anomalous minus expected: *Exp1*: −0.2μV, *Exp2a:* 2.4μV, *t*(58) = 3.54, *p* < .001). In contrast there were no differences between the two experiments in the magnitude of the N400 effect (unexpected minus expected: *Exp1*: −2.6μV, *Exp2a*: −3.1μV, *t*(58) = 0.59, *p* = .56), or the late frontal positivity effect (unexpected minus expected: *Exp1*: 0.6μV, *Exp2a*: 0.2μV, *t*(58) = 0.53, *p* = .60).

##### Comparison between Experiment 2a and Experiment 2b.

Although modulation of the N400 effect and late posterior positivity/P600 effect appeared to be similar between Experiment 2a (locally constraining discourse contexts) and Experiment 2b (globally constraining discourse contexts), we saw clear differences between the two experiments in the modulation of the late frontal positivity. Specifically, in Experiment 2a, no late frontal positivity effect was observed to unexpected versus expected critical words, but this effect was present in Experiment 2b.

**Figure 4.**
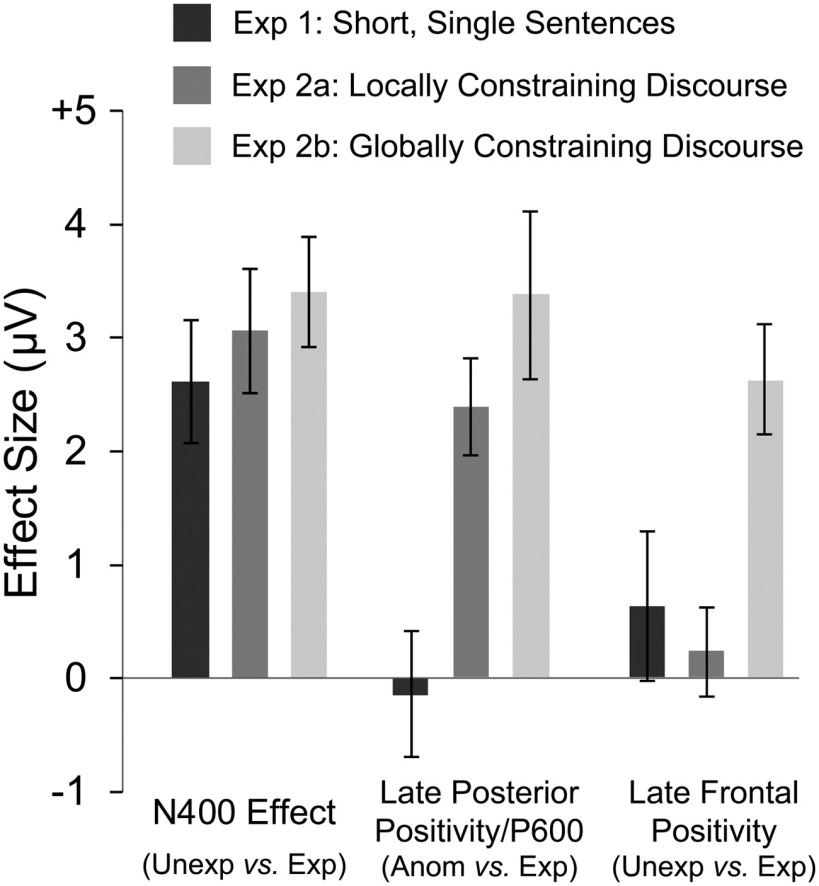
Mean amplitude of the N400 effect (Unexpected minus Expected), the late posterior positivity/P600 effect (Anomalous minus Expected), and the late frontal positivity effect (Unexpected minus Expected) across the three experiments. Error bars represent ±1 standard error of the mean (SEM).

To determine whether these differences were significant, we compared all three effects across the two experiments. This analysis confirmed a significant difference between Experiment 2a and Experiment 2b in the late frontal positivity effect (unexpected minus expected: *Exp2a*: 0.2μV, *Exp2b*: 2.6μV, *t*(29) = 3.78, *p* < .001). In contrast, there were no differences between the two experiments in the magnitude of the N400 effect (unexpected minus expected: *Exp2a*: −3.1μV, *Exp2b*: −3.5μV, *t*(29) = −0.68, *p* = .50), or the late posterior positivity/P600 effect (anomalous minus expected: *Exp2a*: 2.4μV; *Exp2b*: 3.4μV, *t*(29) = 1.16, *p* = .25). See Figures 3 and 4 for a summary of these effects across experiments.

### Discussion

The findings of Experiments 2a and 2b were quite clear. As in Experiment 1, words that were expected produced a smaller N400 response than words that were unexpected. This N400 effect did not differ significantly between Experiment 2a when critical words appeared in locally constraining contexts, and Experiment 2b when critical words appeared in globally constraining contexts.

However, unlike in Experiment 1, the addition of a prior discourse context resulted in clear ERP differences in the post-N400 time-window (600–1,000 ms). It is important to note that the pattern of these late effects differed depending on whether the context was impoverished and locally constraining, or rich and globally constraining. Although a large late posterior positivity/P600 effect was produced by anomalous (vs. expected) words following both locally and globally constraining discourse contexts (i.e., in both Experiments 2a and 2b), a larger late frontal positivity effect was produced by unexpected (vs. expected) words only in rich, globally constraining discourse contexts in Experiment 2b.

In conjunction with the results of Experiment 1, these findings suggest that readers’ engagement in comprehending a prior linguistic context was linked to the production of a late posterior positivity/P600 effect to semantic anomalies. Second, they suggest that the global constraint of the prior context is an important predictor of whether readers will produce a late frontal positivity to unexpected critical words that violate strong lexical predictions.

## GENERAL DISCUSSION

In the present study, we examined online language comprehension while participants read sentences and judged whether or not they made sense. Specifically, we examined neural responses to words that were expected, unexpected, or semantically anomalous, given the constraints of the prior context. In two experiments, we asked whether words that confirmed or violated a comprehender’s prior predictions about a specific upcoming event, or about what is possible in the real world, are processed differently depending on the extent and nature of the preceding linguistic context.

In Experiment 1, participants read very short sentences in which lexical constraint stemmed entirely from a single preceding verb (*James unlocked the…*). Consistent with the prior literature, highly predictable words (*door*) produced a smaller N400 than the other two conditions. However, relative to these expected words, no late frontal positivity effect was produced by unexpected but plausible continuations (*James unlocked the laptop…*), and no late posterior positivity/P600 was produced by semantically anomalous words that violated the selectional constraints of the verb (*James unlocked the gardener…*). These null results differed from those of previous ERP studies, which have reported both of these post-N400 ERP effects in extended, multi-sentence discourse contexts (DeLong et al., 2014; Kuperberg et al., 2020). These findings therefore provided some support for the claim that certain aspects of online language processing proceed differently in impoverished linguistic environments (Kuperberg, 2007; Szewczyk & Schriefers, 2011).

In Experiment 2a, these same locally constraining sentence contexts were presented as part of a longer, three-sentence discourse. It is notable that the first two sentences of these discourse scenarios were relatively semantically impoverished and were not strongly connected to the predicted event in the final critical sentences. Therefore, just as in Experiment 1, the lexical constraint of the context, prior to the critical noun, was determined almost entirely by lexical properties of the preceding verb. We found that, in comparison with Experiment 1, the added discourse context made no difference to the modulation of the N400 or the late frontal positivity. However, within these longer discourse contexts, the semantic anomalies now elicited a robust late posterior positivity/P600 effect. This finding suggests that the elicitation of the late posterior positivity/P600 to semantically anomalous words is associated with the presence of an extended linguistic context.

Finally, in Experiment 2b, participants read a separate set of rich discourse scenarios, which also contained expected, unexpected, or anomalous continuations. In these scenarios, instead of the contextual constraint stemming from a single verb, it was derived from the entirety of a rich, interconnected discourse passage. Just as in Experiments 1 and 2a, we saw effects of contextual constraint on the N400, and, just as in Experiment 2a, we saw a robust late posterior positivity/P600 effect to semantic anomalies. Critically though, this was the only experiment in which we also observed a robust late frontal positivity to the unexpected but plausible continuations.

Taken together, these findings suggest that the comprehension of an extended context played an important role in producing both late positivity effects in the present study. Obviously, we do not wish to claim that multi-sentence discourse contexts are always required to elicit these effects. Several prior studies using extended, single-sentence materials have reported late posterior positivity/P600 effects (e.g., Kuperberg et al., 2003) and late frontal positivity effects (e.g., Federmeier et al., 2007). However, these findings provide strong evidence that both the presence of an extended linguistic context and its semantic richness can influence the elicitation of these two ERP responses.

In the following sections, we discuss the pattern of dissociations among these three ERP components across the three experiments. We then consider how these findings might shed light on the functional underpinnings of the ERP components themselves, as well as the broader theoretical implications of our findings.

### The N400

One robust finding from the present set of studies was the consistent reduction in N400 amplitude to contextually expected critical words. The magnitude and timing of this N400 reduction did not differ depending on whether the constraint of the context stemmed from a single verb (as in Experiments 1 and 2a) or from a rich discourse scenario (as in Experiment 2b). Although these locally and globally constraining contexts were carefully matched in lexical constraint (as determined by cloze ratings), this did not necessarily ensure that we would observe equivalent N400 reductions across contexts. Indeed, there are many examples in the literature of dissociations between offline measures of predictability and N400 amplitudes (e.g., Chow, Smith, Lau, & Phillips, 2016; Urbach & Kutas, 2010; Xiang & Kuperberg, 2015; see Kuperberg, 2016, for discussion), suggesting that, under some circumstances, the full set of information available within a context is not mobilized quickly enough to predict upcoming semantic information or facilitate semantic processing. The fact that no such dissociation occurred in the present data set suggests that sentence boundaries alone do not present a meaningful barrier to anticipatory semantic processing during language comprehension (Fitzsimmons & Drieghe, 2013; Van Berkum et al., 1999). If anything, we saw a small but nonsignificant trend toward greater N400 facilitation effects in the globally constraining discourse contexts (Experiment 2b).

We should note, however, that this pattern may not hold across all communicative environments or in all groups of comprehenders. For example, it has been shown that individuals with reduced working memory capacity show enhanced N400 priming from local, lexical associates (*arms and legs*), even when these associates are incongruent with the global discourse context (Boudewyn, Long, & Swaab, 2013; Van Petten, Weckerly, McIsaac, & Kutas, 1997). Similarly, individuals with schizophrenia show impaired use of global (relative to local) contextual information (e.g., Swaab et al., 2013; see Kuperberg, 2010, for a review). Thus, although the present set of findings suggests that local and global constraints exert similar effects in skilled comprehenders, this equivalency may break down under high levels of processing load and in certain populations.

### The late posterior positivity/P600

Our results have important implications for understanding the functional significance of the “semantic” late posterior positivity/P600. As noted in the General Introduction, this component is often observed in response to words that are anomalous given the constraints of the prior context (e.g., Kuperberg, 2007; Kuperberg et al., 2020; van de Meerendonk et al., 2009). In Experiment 1, however, participants showed no evidence of a late posterior positivity/P600 to semantic anomalies. This mirrors the findings of prior studies that used short, generally nonconstraining sentence contexts (Ainsworth-Darnell et al., 1998; Friederici et al., 1993, 1999; Gunter et al., 1999; Kos et al., 2010; Osterhout & Nicol, 1999). It is notable that in our study, this null effect was found despite participants’ high accuracy in classifying the sentences as anomalous at the end of each trial (see also Gunter et al., 1999; Osterhout & Nicol, 1999). This suggests that merely detecting an anomaly is insufficient to produce the late posterior positivity/P600.

The appearance of the late posterior positivity/P600 in Experiments 2a and 2b suggests that comprehending an extended language context played a key role in triggering this effect. We suggest that this is because prior context motivated comprehenders to construct a situation model of the discourse and to engage in deep comprehension. When new bottom-up information conflicted with constraints of the situation model (i.e., constraints concerning semantic possibility/impossibility), this resulted in a failure to incorporate this new information, triggering a late posterior positivity/P600 (see Kuperberg et al., 2020; Shetreet, Alexander, Romoli, Chierchia, & Kuperberg, 2019). We suggest that this failure, in turn, led the comprehender to engage in second-pass attempts to make sense of the input (see below).

In contrast, when processing the minimal contexts presented in Experiment 1, we suggest that comprehenders failed to establish a situation model at all, and instead processed the surface structure of the text (cf. Graesser, Singer, & Trabasso, 1994; Kintsch & van Dijk, 1978), with the primary goal of detecting the match or mismatch between the thematic structure of the verb and its argument. This interpretation is consistent with previous findings that no late posterior positivity/P600 is produced by syntactic violations in jabberwocky sentences (in which content words were replaced by pseudowords), even though participants were highly accurate in detecting these violations (Ericsson, Olofsson, Nordin, Rudolfsson, & Sandstrom, 2008; Münte, Matzke, & Johannes, 1997).

It is worth noting that these findings are incompatible with theories that attribute the late posterior positivity/P600 simply to a binary task-relevant categorization of whether the critical word is plausible or anomalous (e.g., Bornkessel-Schlesewsky, et al., 2011; see also Sassenhagen, Schlesewsky, & Bornkessel-Schlesewsky, 2014). In Experiment 1, participants carried out the same judgment task as that used in Experiments 2a and 2b, but no hint of this effect was observed at the critical word or subsequent words of the sentence (data not shown). Rather than simply indexing the successful detection of a semantic anomaly, we believe the late posterior positivity/P600 instead reflects the commitment to engage in additional processing of the bottom-up input, in service of the broader goal of successful comprehension. These additional processing stages may involve a reanalysis of the prior context (van de Meerendonk et al., 2010), attempts to repair the prior context, and/or second-pass attempts to come to new representation of meaning (Brouwer, Fitz, & Hoeks, 2012; Kuperberg, 2007; Kuperberg, Caplan, Sitnikova, Eddy, & Holcomb, 2006; Paczynski & Kuperberg, 2012). We return to this point below in Open Questions and Future Directions.

### The Late Frontal Positivity

The present pattern of results also has implications for understanding the functional significance of the late frontal positivity. As noted in the General Introduction, this effect is often observed to words that are plausible but still highly unexpected given the constraints of the prior context (Federmeier et al., 2007; Van Petten & Luka, 2012). One possibility is that the late frontal positivity purely indexes the violation of lexicosemantic constraint. However, the absence of a late frontal positivity in Experiment 1 and Experiment 2a suggests that this ERP effect cannot solely reflect the detection of a lexical prediction violation or activity occurring only at the lexicosemantic level (see also Brothers et al., 2015; Kuperberg et al., 2020; Lau, Holcomb, & Kuperberg, 2013). In both Experiment 1 and Experiment 2a, comprehenders had enough information to predict a specific upcoming word, and this lexical prediction was ultimately violated by the lexicosemantic features of the bottom-up input. However, despite producing differences in the amplitude of the N400, these unexpected words failed to generate a late frontal positivity.

The appearance of a robust late frontal positivity to the unexpected continuations in Experiment 2b suggests that the rich, globally constraining context played a critical role in producing this effect. We suggest that this is because, within these contexts, readers had established a rich situation model into which they had already incorporated a likely upcoming event (e.g., inferring that someone was rolling out dough in a pie-making scenario). When the bottom-up input (e.g., *foil*) was inconsistent with this situation model, it led to a large shift or re-evaluation of this model for the comprehender to come to a new interpretation of the passage as a whole (see Kuperberg et al., 2020). Within such constraining sentence contexts, the completion of this high-level updating process is likely to have entailed top-down suppression of an incorrect lexicosemantic prediction (e.g., the predicted word *dough*, being suppressed as the unexpected target is accessed and selected; see also Ness & Meltzer-Asscher, 2018).

On this account, the reason that no late frontal positivity was produced in Experiment 1 is that the context was so minimal that comprehenders failed to engage in deep comprehension at all—that is, they failed to set up any sort of situation model at the point of the critical word. It was not produced in Experiment 2a because the anticipated event (*unlocking the door*) was not strongly connected to the full set of events that came before. Therefore, at the point of the critical noun, this predicted event had not been fully incorporated (pre-updated) into the prior situation model, and the unexpected continuation (*laptop*) did not trigger a large update at the level of the situation model, relative to the expected continuation.

### Theoretical Implications

Several researchers have argued that the N400 primarily reflects the effects of confirmed semantic predictions, whereas the late positivities are associated with prediction violations. Moreover, it has been noted that the topography of these late components (frontal vs. posterior) is linked to whether the resulting interpretation of the unexpected input is semantically plausible or anomalous (Kuperberg et al., 2020; Van Petten & Luka, 2012). The present findings build upon these observations by suggesting that offline measures of lexical probability and plausibility, when viewed in isolation, cannot provide a full account of comprehenders’ online neural responses to predictable and unpredictable content during comprehension.

Instead, these findings provide strong evidence for the idea that the late posterior positivity/P600 cannot simply reflect the detection of a semantic anomaly, and that the late frontal positivity cannot simply reflect the detection of a lexical prediction violation. Instead, these findings demonstrate that an extended prior context plays an important role in eliciting these effects. We argue that this is because, by comprehending these contexts, readers established a situation model. We suggest that both late positivity effects reflect activity at this high level of representation.

**Figure 5.**
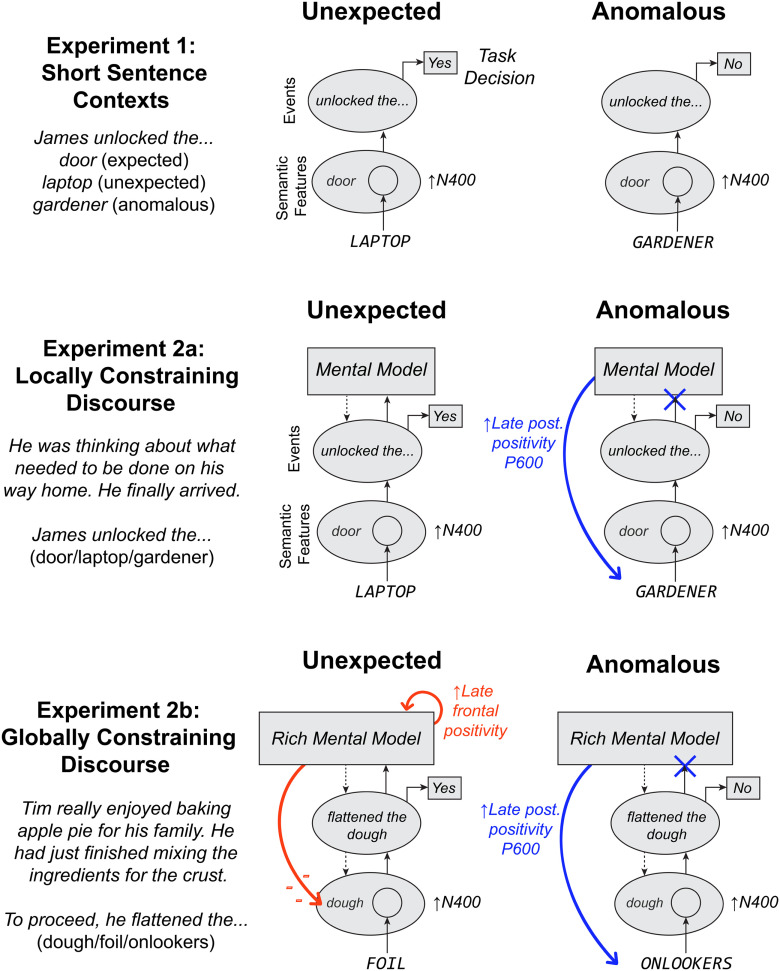
A schematic diagram illustrating differences in the structure and state of reader’s hierarchical generative networks across experiments and conditions. Dotted arrows represent anticipatory information that has been passed down through the hierarchy prior to word onset, while solid, upward arrows represent bottom-up information being passed from the lexical input through higher levels of the hierarchy. Increased N400 responses are thought to arise at the semantic feature level, as new unpredicted semantic information is accessed/decoded. The two late positivities are thought to arise when new information leads to large updates in the prior situation model (late frontal positivity, red), or when information conflicts with the constraints of the communication model, triggering re-analysis/repair (late posterior positivity/P600, blue).

We next argue that the full set of findings can be understood within a hierarchical generative framework of language processing (see also, Kuperberg, 2016; Kuperberg & Jaeger, 2016; Kuperberg et al., 2020). As noted in the General Introduction, within this framework, the particular subset of stored linguistic and nonlinguistic knowledge that an agent brings to any language task—their *hierarchical generative model—*will depend on their overall communicative goal (see Figure 5).

We suggest that in Experiment 1, when the prior context was extremely short, participants assembled a generative model that constituted the minimal number of levels required to fulfill the requirements of the task—that is, to decide whether or not each sentence made sense. The model included levels of representation that encoded the semantic and syntactic features of verbs and nouns, which were combined into event structures defining the broad thematic-semantic roles of “who does what to whom.” Critically, this generative network did not include a still higher level of representation that encoded a situation model.

We suggest that, in Experiment 1, information was passed up the network primarily in a bottom-up fashion. Thus, constraints at the verb preactivated fine-grained semantic properties, as well as the part of speech of the upcoming noun phrase. Together, these allowed for the bottom-up anticipation of a specific upcoming event structure (e.g., *Agent unlocked inanimate Patient*). Expected critical nouns (e.g., *unlocked the door*) matched both fine-grained semantic predictions as well as the predicted event structure. Unexpected critical nouns mismatched the predicted semantic features, producing a larger N400, but were consistent with the verb’s predicted event structure. Anomalous critical nouns again mismatched the predicted semantic features producing a larger N400, and they also mismatched the predicted event structure. Being able to distinguish between the match or mismatch at the level of the event structure was sufficient for participants to meet the requirements of the task. Crucially, because there was no high-level situation model, no late positivities were produced.

In contrast, we argue that in Experiments 2a and 2b, participants engaged in a deeper mode of comprehension, with the implicit goal of establishing coherence within and across sentences (Graesser et al., 1994). To accomplish this goal, they assembled a generative network that included not only semantic, syntactic, and event structure representations, but also a still higher level of representation that encoded the situation model. This complete communication model established the constraints of the situation model—the assumption that the communicator would convey events that were possible in light of the preceding context, while ruling out potential event structures that conveyed impossible events (e.g., *unlocking a gardener*).

In both Experiments 2a and 2b, in the anomalous condition, the bottom-up input conflicted with the constraints of the situation model, leading to an initial failure to incorporate this input and a temporary arrest in comprehension. However, because, within this generative framework, the overarching goal is to explain the bottom-up input, the comprehender likely attempted to resolve this conflict through a second-pass analysis—that is, reanalyzing or repairing the input, and/or revising the communication model itself. We suggest that the late posterior positivity/P600 reflected this decision to engage in second-pass attempts to explain the input.

In Experiment 2b, participants not only established a general situation model, but, prior to encountering the incoming word, they had established a rich and interconnected situation model, into which they had already incorporated a specific upcoming event. When the unexpected, bottom-up input reached this top layer of the hierarchy, it led to a shift/update in the participants’ prior interpretation. We suggest that the late frontal positivity reflected this large shift in the situation model to reach a new coherent interpretation.

### Open Questions and Future Directions

Our findings highlight a number of important questions for future research. With regard to the late frontal positivity, they suggest that rich globally constraining contexts play an important role in eliciting this effect following a prediction violation. Critically though, there are several examples in the literature of enhanced late frontal positivities to unexpected words, even within lexically nonconstraining sentence contexts (Federmeier & Kutas, 2005; Thornhill & Van Petten, 2012; Zirnstein, van Hell, & Kroll, 2018). These findings can be explained within a hierarchical generative framework if it is assumed that, in such cases, unexpected incoming words are highly informative, producing a large update in the comprehenders’ situation model, even though no specific event had been previously predicted. This hypothesis could be tested more directly in future studies by comparing ERP responses to contextually informative and uninformative words while holding other factors, such as cloze probability and lexical constraint, constant.

A second set of questions concerns the precise functional role of the late posterior positivity/P600. Here we have argued that the key trigger for this effect, at the point of the critical word, is not simply a dichotomous decision or classification of sentences as well-formed or semantically anomalous (Bornkessel-Schlesewsky et al., 2011; Sassenhagen et al., 2014), but rather the downstream consequences of this decision. This has some interesting parallels with analogous discussions about the functional significance of the well-known P300 ERP component (Donchin & Coles, 1988), which shares common computational principles with the late posterior positivity/P600 (Coulson, King, & Kutas, 1998; Osterhout, Kim, & Kuperberg, 2012; Sassenhagen et al., 2014). Although the posterior P300 is classically produced when participants make categorical task-relevant decisions about an eliciting event, it has been argued that simply making a task-relevant categorical decision is not sufficient to produce this effect; rather, what is critical is that this decision must have some utility for future action, described broadly by Donchin as “context updating” (Donchin & Coles, 1988). This commitment to engage in this future-oriented activity (contextual updating) may be linked to the top-down allocation of attention, functioning to increases the gain on information processing, and possibly mediated by the phasic release of the neuromodulator norepinephrine (Nieuwenhuis, Aston-Jones, & Cohen, 2005; Yu & Dayan, 2005). In future studies, it will be important to test whether the late posterior positivity/P600 elicited to anomalous inputs during language comprehension is similarly linked to this type of top-down modulation of attentional gain.

Finally, the findings of this study tie into a large literature suggesting that comprehenders can approach a text with different goals and “standards of coherence,” depending on task demands and their own internal motivations (Graesser et al., 1994; van den Broek, Bohn-Gettler, Kendeou, Carlson, & White, 2011). This has important implications for understanding the neurocognitive basis of poor reading comprehension. Skilled comprehenders are able to monitor and detect breaks in coherence during online processing, engaging in reanalysis and repair strategies to prevent comprehension failures, while poor readers are less accurate at detecting these coherence breaks and are less able to compensate accordingly (Garner, 1980; McInnes, Humphries, Hogg-Johnson, & Tannock, 2003; Wagoner, 1983). It will be important for future studies to investigate whether these two late positivities might provide a useful neural marker for probing these coherence monitoring processes during online language comprehension.

## ACKNOWLEDGMENTS

The Sidney R. Baer Jr. Foundation supported undergraduate students Rebecca Nardulli and Simone Riley, who contributed to data collection. We would also like to thank Maria Luiza Cunha Lima and Bram Vandekerchkove for their contributions in constructing the experimental materials; Minjae Kim, Connie Choi, and Sophie Greene for their assistance with data collection; and Arim Choi and Lin Wang for helpful feedback and comments in preparing the manuscript.

## AUTHOR CONTRIBUTIONS

Trevor Brothers: Conceptualization; Methodology; Formal analysis; Writing – original draft; Review & editing. Eddie W. Wlotko: Conceptualization; Methodology; Funding acquisition. Lena Warnke: Conceptualization; Methodology; Review & editing. Gina R. Kuperberg: Conceptualization; Methodology; Review & editing; Funding acquisition; Supervision.

## FUNDING INFORMATION

G. R. Kuperberg, National Institute of Child Health and Human Development (http://dx.doi.org/10.13039/100000071), Award ID: R01 HD08252.
